# Predictive nomogram for coronary heart disease in patients with type 2 diabetes mellitus

**DOI:** 10.3389/fcvm.2022.1052547

**Published:** 2022-11-10

**Authors:** Shucai Xiao, Youzheng Dong, Bin Huang, Xinghua Jiang

**Affiliations:** Department of Cardiovascular Medicine, The Second Affiliated Hospital of Nanchang University, Nanchang, China

**Keywords:** prediction model, nomogram, coronary heart disease, type 2 diabetes mellitus, coronary angiography

## Abstract

**Objective:**

This study aimed to identify risk factors for coronary heart disease (CHD) in patients with type 2 diabetes mellitus (T2DM), build a clinical prediction model, and draw a nomogram.

**Study design and methods:**

Coronary angiography was performed for 1,808 diabetic patients who were recruited at the department of cardiology in The Second Affiliated Hospital of Nanchang University from June 2020 to June 2022. After applying exclusion criteria, 560 patients were finally enrolled in this study and randomly divided into training cohorts (*n* = 392) and validation cohorts (*n* = 168). The least absolute shrinkage and selection operator (LASSO) is used to filter features in the training dataset. Finally, we use logical regression to establish a prediction model for the selected features and draw a nomogram.

**Results:**

The discrimination, calibration, and clinical usefulness of the prediction model were evaluated using the c-index, receiver operating characteristic (ROC) curve, calibration chart, and decision curve. The effects of gender, diabetes duration, non-high-density lipoprotein cholesterol, apolipoprotein A1, lipoprotein (a), homocysteine, atherogenic index of plasma (AIP), nerve conduction velocity, and carotid plaque merit further study. The C-index was 0.803 (0.759–0.847) in the training cohort and 0.775 (0.705–0.845) in the validation cohort. In the ROC curve, the Area Under Curve (AUC) of the training set is 0.802, and the AUC of the validation set is 0.753. The calibration curve showed no overfitting of the model. The decision curve analysis (DCA) demonstrated that the nomogram is effective in clinical practice.

**Conclusion:**

Based on clinical information, we established a prediction model for CHD in patients with T2DM.

## Introduction

According to the International Diabetes Federation, 537 million people aged 20–79 are estimated to have diabetes, and 783 million are expected to have diabetes by 2045 (1). Recent studies have shown that diabetes is associated with a 75% increase in cardiovascular mortality in adults. (2). Coronary heart disease (CHD) is one of the most common cardiovascular complications in diabetes patients. CHD accounts for 65% of deaths in diabetes patients (2, 3). The risk of CHD is twice higher in diabetic patients than in non-diabetes individuals (4). Chronic hyperglycemia exacerbates atherosclerosis plays an important role in the development of cardiovascular disease (5). The main mechanisms may be endothelial cell dysfunction, increased advanced glycation end products (AGEs), and severe oxidative stress induced by chronic hyperglycemia (6). Diabetes mellitus is one of the established risk factors for cardiovascular disease. Therefore, it is necessary to accurately identify the high-risk factors and high-risk groups of CHD in patients with type 2 diabetes mellitus (T2DM) to prevent CHD.

Currently, coronary angiography is the gold standard for the diagnosis of CHD. However, it is an invasive method and not directly applicable to some patients (e.g., individuals with contrast agent allergy or claustrophobia). Thus, the objective of this study is to develop a prediction model and identify patients with T2DM who are at high risk of CHD. By using this model, a subset of unnecessary coronary angiography can be avoided. In recent years, the nomogram has become a commonly used predictive tool. The nomogram is a convenient tool to draw in the same plane according to a certain scale using a line with a scale. Its interpretation and usage are easier for medical staff. It can be conveniently and rapidly used in the clinical setting. The development and application of non-invasive systems to screen patients at high risk for early CHD is key for primary prevention of CHD.

## Participants and methods

This study was designed as a retrospective study based on The Second Affiliated Hospital of Nanchang University medical record system. In this study, 1,808 T2DM patients hospitalized for coronary angiography (CAG) from June 2021 to June 2022 were included. Five hundred sixty patients were prospectively enrolled after screening for inclusion and exclusion criteria. Of these, 70% were randomly assigned to the training cohort (392 cases), and the remaining cases were included in the validation cohort (168 cases). The purpose of setting the training cohort was to build a model. The validation cohort was set up to confirm the performance of the model. Patients were not eligible for inclusion if the following exclusion criteria were met: (1) History of cardiovascular heart diseases, including myocardial infarction or revascularization procedures. (2) Patients without complete clinical data. (3) Age less than 18 years old.

## Data collection

Relevant demographic variables, including age, gender, body mass index (BMI), and history of hypertension, were collected for all patients. Fasting blood samples were collected in the morning for laboratory tests, including fasting blood glucose (FBG), glycosylated hemoglobin (HbA1C), serum uric acid (Uric), total bilirubin lipids (TBIL), low-density lipoprotein cholesterol (LDL-C), high-density lipoprotein cholesterol (HDL-C), Remnant cholesterol (RC), non-high-density lipoprotein cholesterol (Non-HDL-C), creatinine (CRE), total cholesterol (TC), triglycerides (TG), apolipoprotein A1 (APOA1), apolipoprotein B (APOB), lipoprotein-a [Lp(a)], homocysteine (HCY), and total cholesterol (TC). Atherogenic index of plasma (AIP) and triglyceride-glucose index (TYG) were calculated using the following formula. The AIP index was calculated as log (triglyceride/high-density lipoprotein cholesterol). The TyG index was determined using ln(fasting triglycerides [mg/dL] × fasting glucose [mg/dL]/2). At the same time, the examination results of nerve conduction velocity (NCV), color ultrasound of the carotid artery, and coronary angiography were recorded.

## Statistical analysis

The statistical analysis was conducted using R software (version 4.2.1)^1^ and SPSS 25.0. First, data from all patients were collected to establish the dataset, which was randomly split into training and validation datasets in a 3:1 ratio by R software. Data are expressed as mean values and standard deviation (SD), and analyzed using SPSS. Continuous variables were compared using two-tailed *t*-test or Mann–Whitney *U* test, depending on data distribution. Categorical variables are expressed as percentages and numbers and compared using the chi-square test. Then, the features were screened using least absolute shrinkage and selection operator (LASSO) regression analysis on the data from the training dataset.

Least absolute shrinkage and selection operator is a well-known method for regression with high-dimensional data. After filtering out features with non-zero coefficients, odds ratio (OR) with corresponding 95% confidence interval (95% CI) were calculated for each factor using multivariate logistic regression. Then obtain the corresponding *P*-values of each factor. The statistical significance levels were all two-sided. We selected risk factors with a *P*-value of <0.05 based on logistic regression to construct a predictive model and draw nomograms. We used R software for the training and validation data sets to calculate the C-index and draw receiver operating characteristic (ROC) curves, calibration curves, and performed decision curve analysis (DCA) to test the accuracy of the prediction model.

## Results

### Baseline characteristics

Between June 2021 and June 2022, 1808 T2DM patients who underwent coronary angiography (CAG) at our hospital were included in our study. Following the screening, 560 participants who had complete data were included in this study. Of them, 326 were patients diagnosed with CHD. The whole patient cohort was randomly divided into a training cohort containing 392 (70%) patients and a validation cohort including 168 (30%) patients. The demographic and clinical characteristics of the training and validation cohorts are presented in Table 1. In the training and validation cohorts, the proportion of patients with confirmed CHD was 59.2 and 56%, respectively. At baseline, there were no significant differences in the distribution of demographic and clinical characteristics between the two cohorts (Table 1).

**TABLE 1:** Characteristics of the patients in different cohort.

Variables	Training cohort	Validation cohort	*P*
	(*n* = 392)	(*n* = 168)	
Age, year	63.72 ± 9.956	64.45 ± 10.363	0.477
Male, sex	221 (56.38%)	103 (63.31%)	0.279
BMI ≥24	24.90 ± 3.246	25.57 ± 3.717	0.088
Hypertension	272 (69.39%)	134 (79.76%)	0.012
DM duration ≥10 year	164 (41.84%)	66 (39.29)	0.316
HbA1c, mmol/	7.86 ± 4.932	7.58 ± 1.730	0.45
Uric, μmol/l	362.17 ± 112.552	380.61 ± 116.685	0.058
Cre, μmol/l	94.55 ± 103.675	88.39 ± 42.049	0.236
TBIL, μmol/L	13.02 ± 5.923	13.21 ± 6.114	0.806
TG, mmol/l	2.14 ± 1.831	2.41 ± 2.174	0.183
TC, mmol/l	4.75 ± 1.367	4.79 ± 1.329	0.49
HDL-C, mmol/l	1.10 ± 0.316	1.11 ± 0.349	0.669
LDL-C, mmol/l	2.87 ± 1.075	2.92 ± 1.119	0.702
Non-HDL-C, mmol/l	3.64 ± 1.321	3.69 ± 1.339	0.612
RC, mmol/l	0.77 ± 0.423	0.77 ± 0.582	0.822
APOA1, g/l	1.279 ± 0.305	1.286 ± 0.323	0.898
APOB, g/l	1.01 ± 0.355	1.03 ± 0.362	0.359
Lp(a), mg/l	255.64 ± 322.691	229.76 ± 301.879	0.592
HCY, μmol/L	13.23 ± 5.390	15.12 ± 11.195	0.019
FBG, mmol/l	9.25 ± 4.135	9.43 ± 4.730	0.841
TYG	9.364 ± 0.828	9.445 ± 0.949	0.443
AIP	0.205 ± 0.346	0.247 ± 0.374	0.19
NCV slow	146 (37.24%)	56 (33.33%)	0.377
Carotid plaque	318 (81.12%)	135 (80.36%)	0.833

BMI, body mass index; DM, diabetes mellitus; HbA1C, glycosylated hemoglobin; Uric, serum uric acid; CRE, creatinine; TBIL, total bilirubin lipids; TG, triglycerides; TC, total cholesterol; HDL-C, high-density lipoprotein cholesterol; LDL-C, low-density lipoprotein cholesterol; Non-HDL-C, non-high-density lipoprotein cholesterol; RC, remnant cholesterol; APOA1, apolipoprotein A1; APOB, apolipoprotein B; Lp(a), lipoprotein-a; HCY, homocysteine; FBG, fasting blood glucose; TYG, triglyceride-glucose index; AIP, atherogenic index of plasma; NCV slow, nerve conduction velocities were slowed.

### Feature selection

**FIGURE 1:**
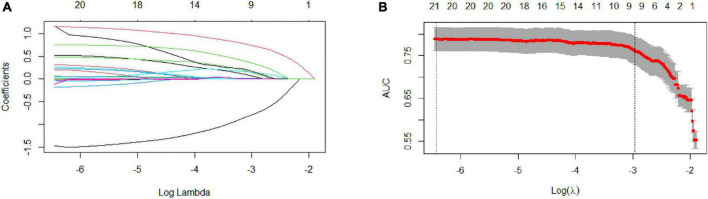
Selection of coronary heart disease (CHD) risk factors using the LASSO regression method. **(A)** In the LASSO model, tuning parameter (λ) selection was performed with 10-fold cross-validation *via* minimum criteria. The Area Under Curve (AUC) curve is plotted against log (λ). Dotted vertical lines are drawn at the optimal values, as determined using the minimum criteria and 1 standard error of the minimum criteria (1-SE criteria). **(B)** LASSO coefficient profiles of the 24 texture features. A coefficient profile plot is produced against the log (λ) sequence. A vertical line is drawn at the value chosen using 1000-fold cross-validation, where optimal l resulted in 9 non-zero coefficients. LASSO, least absolute shrinkage and selection operator; SE, standard error.

After LASSO analysis, 24 features were simplified into 9 potential predictors with non-zero coefficients based on training data set (Figure 1).

### Nomogram development

**FIGURE 2:**
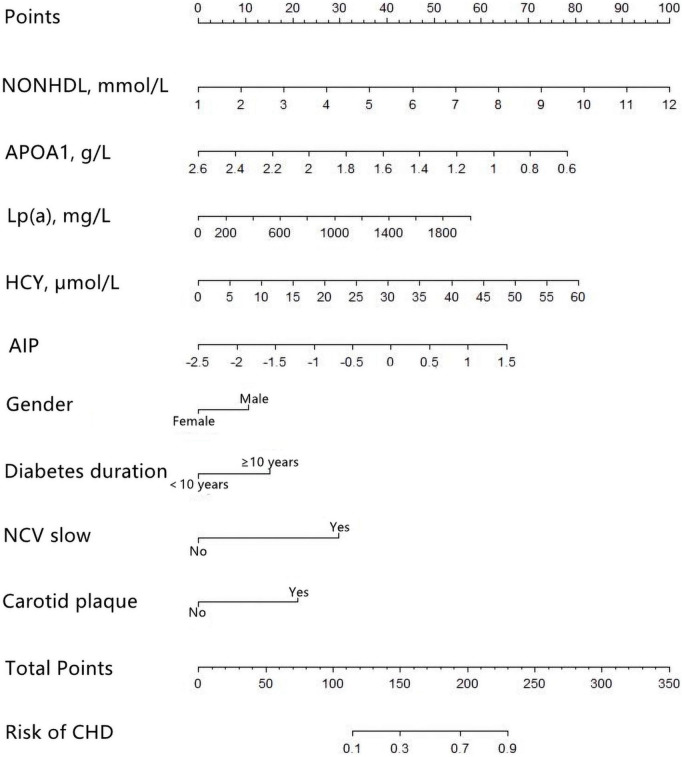
Development of the coronary heart disease (CHD) prediction nomogram. The CHD prediction nomogram was developed in the training cohort, with Gender, Duration, non-high-density lipoprotein cholesterol (non-HDL-C), apolipoprotein A1 (APOA1), lipoprotein-a [Lp(a)], homocysteine (HCY), atherogenic index of plasma (AIP), nerve conduction velocity (NCV), and Carotid plaque as predictors.

Multivariate logistic regression revealed that gender, diabetes duration, non-HDL-C, APOA1, Lp(a), HCY, AIP, NCV, and carotid plaque were the key predictors of CHD in patients with T2DM (Table 2). The nomogram is constructed and drawn based on the above 9 prediction factors (Figure 2).

**TABLE 2:** Model established by logistic regression analysis based on the training cohort.

Prediction factors	β	OR	OR 95% CI	*P*
Gender	0.406	1.501	0.914–2.466	0.109
DM duration	0.581	1.787	1.098–2.91	0.02
Non-HDL-C	0.347	1.414	1.121–1.785	0.003
APOA1	−1.493	0.225	0.089–0.569	0.002
Lp(a)	0.001	1.001	1–1.002	0.019
HCY	0.051	1.053	0.999–1.109	0.054
AIP	0.624	1.866	0.82–4.228	0.135
NCV slow	1.138	3.119	1.847–5.266	<0.001
Carotid plaque	0.803	2.232	1.21–4.116	0.01
Intercept	−1.474			

β is the regression coefficient.

### Nomogram performance

**FIGURE 3:**
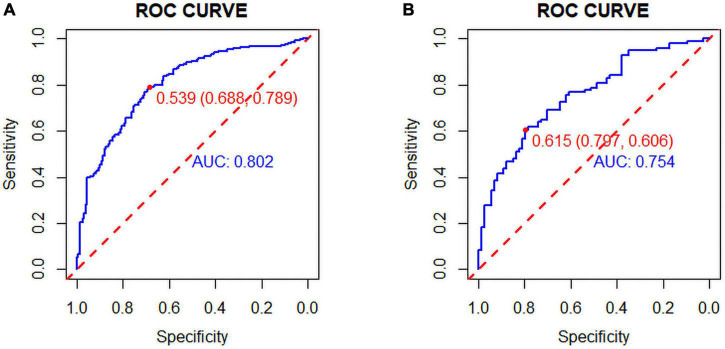
**(A)** Training set and **(B)** Validation set: The pooled Area Under Curve (AUC) of the receiver operating characteristic (ROC) curve. The y-axis indicates the true positive rate of the risk prediction. The x-axis indicates the false positive rate of the risk prediction. The blue and red lines represent the performance of the nomogram.

**FIGURE 4:**
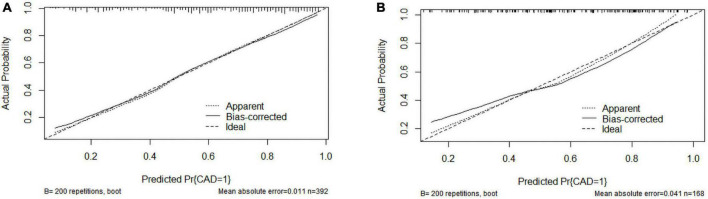
Calibration curves of the coronary heart disease (CHD) prediction nomogram. **(A)** Calibration curve of the CHD prediction nomogram in the training cohort; **(B)** Calibration curve of the CHD prediction nomogram in the validation cohort.

The C-index was 0.803 (0.759–0.847) in the training cohort and 0.775 (0.705–0.845) in the validation cohort. In the ROC curve, the Area Under Curve (AUC) of the training set (Figure 3) is 0.802, and the AUC of the validation set (Figure 3) is 0.753. The calibration curve showed no overfitting of the model (Figure 4). The logistic regression model fitted well with the data (Hosmer–Lemeshow test, *P* = 0.687). The above results proved that this nomogram could predict CHD effectively.

### The clinical application of the nomogram

**FIGURE 5:**
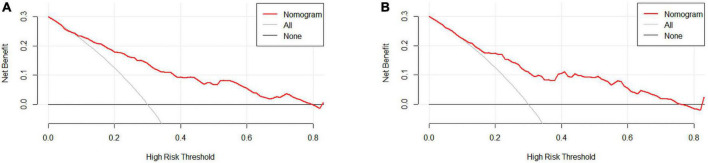
**(A)** Training set and **(B)** Validation set. Decision curve analysis (DCA) for the coronary heart disease (CHD) prediction nomogram. The y-axis measures net benefit. DCA shows the clinical usefulness of the nomogram score, according to a continuum of potential thresholds for CHD risk (x-axis) and the net benefit of using the nomogram to stratify patients according to risk (y-axis). Weighting factor = threshold probability/(1-threshold probability). Net benefit = true positive rate–(false positive rate × weighting factor). The red line represents the incidence risk nomogram of CHD. The gray line represents the assumption that all patients are diagnosed with CHD. The thin thick solid line represents the assumption that no patients are diagnosed with CHD.

We used DCA to evaluate the clinical application of nomogram and compare the net benefits of nomogram and reference models (Figure 5). In most reasonable threshold probability ranges, the DCA showed that the nomogram was clinically useful.

## Discussion

A nomogram is a two-dimensional graph that provides a graphical representation of mathematical relationships or formulas and can be used to calculate the risk of diseases without using a calculator. Nomograms are user-friendly digital interfaces and are accurate in improving clinical decision-making.

Currently, studies have mainly focused on the diagnostic markers or biomarkers of CHD, mainly uncommon and expensive biochemical indicators. In this study, we used the test results from Chinese patients with type 2 diabetes needed when applying for medical insurance. Previous relevant studies on CHD were used as evidence to filter the corresponding variables. These variables included age, gender, DM duration, hypertension, BMI, FBG, HbA1c, blood uric acid, TBIL, TG, TC, HDL-C, LDL-C, NONHD, RC, APOA1, APOB, Lp(a), HCY, serum creatinine, NCV, and carotid plaque.

Through LASSO and logistic regression, our study revealed that gender, diabetes duration, non-HDL-C, APOA1, Lp(a), HCY, AIP, NCV, and carotid plaque were the key predictors of CHD in patients with T2DM. We developed a nomogram involving nine variables to predict the risk of CHD in patients with T2DM.

**FIGURE 6:**
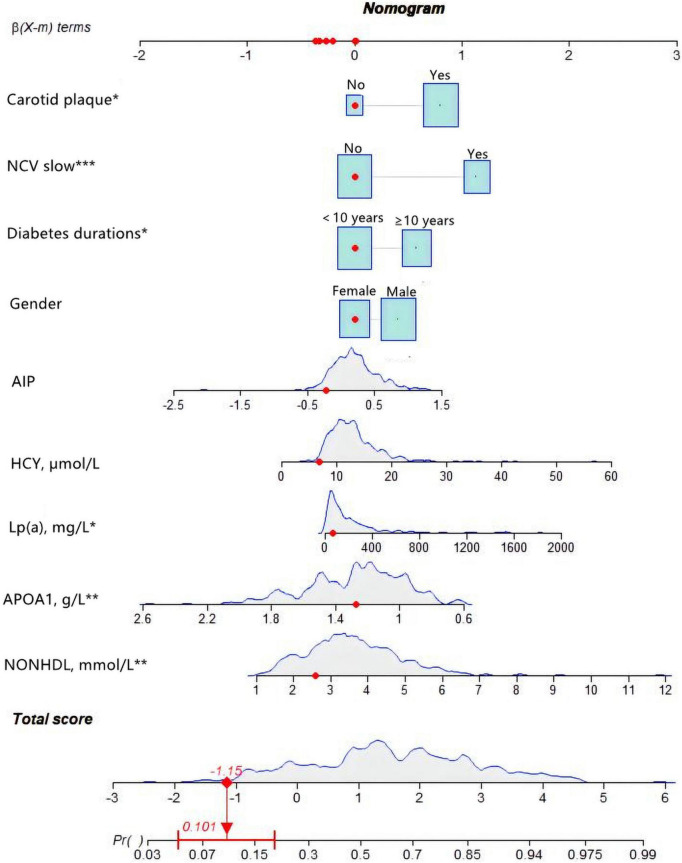
An example of nomogram for coronary heart disease (CHD) in type 2 diabetes mellitus (T2DM) patients. Logistic regression results showed that there were corresponding *P*-values for each index, and the indicators with statistical significance level *P* ≤ 0.05 were included in the nomogram. “***” means *P* < 0.001, “**” means *P* < 0.01, “*” means *P* < 0.05.

For female subjects with T2DM, the DM duration was less than 10 years, non-HDL-C was 2.59 mmol/L, APOA1 was 1.2 mmol/l, Lp(a) was 63.7, HCY was 6.85 mmol/l, and AIP index was −0.2253. With normal nerve conduction velocity and no carotid plaques found on carotid ultrasound, the probability of coronary heart disease is 10.1% (Figure 6).

A systematic literature review comprising 10 articles from 2000 to 2017 analyzed 5012 T2DM patients without chest pain who underwent CAG or CTA (7). Among them, 33.7% were diagnosed with CHD. In this study, the prevalence of CHD was 58% in all patients. Compared with similar studies, CHD prevalence was slightly higher in this study, possibly due to the inclusion of both symptomatic and asymptomatic patients.

In non-diabetic subjects, CHD is more common in men than in women. However, in this study, there was no significant disparity in the diabetic population. Numerous studies indicated that T2DM increases cardiovascular risk in women more than in men (8, 9).

Although mineralocorticoid receptor activation is an important contributor to impaired vascular function in diabetes, angiotensin-II-stimulated aldosterone release is increased in diabetic women compared with men, exposing women to aldosterone overexposure, which may be one of the mechanisms underlying the excess cardiovascular morbidity in diabetic women (10, 11).

This study revealed a high association between T2DM duration and CHD risk. Many studies have shown that the duration of diabetes is closely related to CHD (11, 12). A Korean single-center retrospective cohort with 2,006 patients revealed that diabetes with a duration of more than 10 years is a common risk factor for CHD (13).

In the present study, high non-HDL-C was a risk factor for CHD in patients with T2DM and was positively associated with the probability of CHD. Non-HDL was calculated as serum total cholesterol minus HDL levels. At present, more and more evidence shows that non-HDL-C is superior to LDL-C in predicting coronary heart disease risk (14, 15). According to the 2019 ESC/EAS guidelines, non-HDL-C should be considered the first indicator when assessing cardiovascular risk in patients with diabetes (16). The possible mechanism is that LDL-C can be affected by apolipoprotein B cholesterol level, while non-HDL-C can reflect apolipoprotein B-related cholesterol level and is related to HDL level (17).

Apolipoprotein A1 is the main protein of high-density lipoprotein (HDL) and has cardiovascular protective properties (18). HDL-C is considered the “good cholesterol” and higher HDL-C levels are correlated with cardiovascular health (19).

However, recent randomized clinical trials of HDL-C enhancers have failed to reduce cardiovascular events (20). Although HDL-C has been recognized as a traditional protective factor against atherosclerosis, not all HDL subsets are equally cardiovascular protective (21). It was found that the protective effect of HDL-C particles on atherosclerosis was impaired in homozygous and heterozygous apoA-I deficiency (22). This may suggest that apoA1 may be a more reliable predictor of CHD than HDL-C (23).

In our study, lipoprotein (a) was a risk factor for CHD in patients with T2DM and was positively associated with the probability of CHD. High lipoprotein (a) levels are associated with an increased risk of ischaemic CVD, particularly CHD (24, 25). Lp(a) is a complex produced in the liver by apolipoprotein (a) linked with disulfide bonds to LDL-C-like particles. Lp(a) contains not only all the atherogenic factors of LDL-C but also one molecule of Apo(a). Apo (a) is structurally similar to plasminogen and has a thrombogenic effect (26). The AIM-HIGH trial revealed that patients with LDL-C up to 65.2 mg/dL (1.62 mmol/L) and Lp(a) >50 mg/dL had a 90% higher risk of major adverse cardiovascular events than patients with similar LDL-C levels but Lp(a) levels below 50 mg/dL (24). A retrospective study of 3213 patients in a Chinese population also suggested that Lp(a) is an independent risk factor for CHD (27).

In the present study, HCY was a risk factor for CHD in patients with T2DM and was positively associated with the probability of CHD. The mechanism is unclear and controversial. The first view is that HCY has a direct cytotoxic effect on the vascular endothelium (28), which can cause vascular endothelial cell damage and functional changes and induce endothelial cell apoptosis (29). The second view is that HCY can affect coagulation and fibrinolysis, thereby promoting thrombosis (29). High levels of HCY can increase the risk of thrombosis, oxidative stress, and endothelial dysfunction.

Some studies have found elevated HCY levels to be a risk factor for CHD (30).

In our study, the value of the AIP index was positively associated with the probability of developing CHD. The concept of AIP was first proposed by Dobiásová and Frohlich (31) as a marker of atherosclerosis. Many studies have reported that elevated AIP is a potential biomarker for predicting cardiovascular diseases (32, 33). Previous studies have shown that both high-levels of TG and low-levels of HDL-C are important markers of CVD. High levels of TG damage the vascular endothelium (34), leading to endothelial dysfunction, coagulation, and activation of inflammatory responses. These alterations contribute to atherogenesis. In contrast, HDL-C plays a major role in reverse cholesterol transport and protects the heart against inflammation and oxidative stress. Many studies have found that AIP is closely related to LDL-C particle size (35, 36), and higher AIP is associated with smaller LDL particles. Small dense LDL-C has been shown to be very susceptible to oxidative damage, which in turn contributes to the development of atherosclerosis (37).

In the present study, decreased nerve conduction velocities were a risk factor for CHD in patients with T2DM. Nerve conduction velocities mentioned in this study were an exam item used to diagnose peripheral neuropathy. Decreased conduction velocities are suggestive of peripheral neuropathy (PN). Cardiac autonomic neuropathy (CAN) is one of the common complications in patients with type 2 diabetes mellitus. Previous studies showed that CAN contributes to increased cardiovascular risk (38). A US study involving 7,116 individuals found a significant association between all-cause and cardiovascular mortality and PN in subjects with diabetes (39). The relationship between CAN and PN has been intensively studied and shown by some studies (40, 41), demonstrating that PN in patients with T2DM can be used as a predictor of CHD.

In the present study, the presence of carotid plaques was a risk factor for CHD in patients with T2DM. Previous reports from the multi-ethnic study of atherosclerosis (MESA) have suggested that the presence of carotid plaques is associated with cardiovascular risk in the entire cohort (42, 43). Recent studies have shown that carotid plaques are independently associated with incident CHD in participants (44). However, intima–media thickness (IMT) was not included in this study since a high variability was observed in the results. The measurements of IMT are mainly based on the personal experience of clinicians with different levels of expertise. A meta-analysis showed that the risk assessment of cardiovascular events did not improve when IMT was added to the Framingham risk score (45).

In contrast to earlier nomogram studies, most of these studies focused on demographic factors associated with CHD (46). Some nomogram studies often obscure the classification of patients into normal and abnormal status without focusing on their specific values. For example, some studies simply classified patients into hyperlipidemic and non-hyperlipidemic groups (47). We used blood lipid, nerve conduction velocity, carotid artery plaque, and other results from medical insurance tests in Chinese patients with type 2 diabetes. These results are easy to obtain from patients, accessible, and stable. It is more applicable to non-cardiovascular physicians to judge the cardiovascular risk of diabetes mellitus patients. Some studies do not mention whether patients undergo coronary angiography (48). The patients in our study had well-established CAG, and the diagnosis was consolidated.

There are some limitations in our study. Firstly, the data were obtained from patients with T2DM who underwent CAG and there may be a selective bias. Second, our risk prediction model was developed from a single center data and lacks external validation. However, we set up separate internal and external validation sets. Third, because of missing data, classical risk factors such as smoking history and drinking history were not included in this study. More studies are needed in the future to further confirm our findings. Finally, only clinical factors were included in this study, and more non-clinical factors could be involved.

## Conclusion

By collecting various information and biochemical test results of patients with T2DM in The Second Affiliated Hospital of Nanchang University and analyzing the relevant data, we built a prediction model for the risk of CHD in patients with T2DM and plotted nomograms. Gender, T2DM duration, Non-high-density lipoprotein cholesterol, apolipoprotein A1, lipoprotein(a), homocysteine, atherogenic index of plasma (AIP), nerve conduction velocity, and carotid plaque were included in the model. Through a variety of statistical methods, it is verified that the nomogram can predict the risk of CHD in T2DM patients with medium accuracy.

## Data availability statement

The original contributions presented in this study are included in the article/supplementary material, further inquiries can be directed to the corresponding author.

## Ethics statement

This study was approved by the Institutional Review Board of the Second Affiliated Hospital of Nanchang University. Written informed consent was acquired from all participants for their participation in our study.

## Author contributions

SX and XJ designed the study. SX and BH collected and analyzed the data. SX and YD wrote the manuscript. All authors read and approved the final manuscript.

## Footnotes

1
https://www.r-project.org
